# Postoperative surgical complications of lymphadenohysterocolpectomy

**Published:** 2014-03-25

**Authors:** F Marin, M Pleşca, CI Bordea, SC Voinea, I Burlănescu, E Ichim, CG Jianu, RR Nicolăescu, MP Teodosie, K Maher, A Blidaru

**Affiliations:** Department of Surgical Oncology II, " Prof. Dr. Al Trestioreanu " Institute of Oncology, Bucharest

**Keywords:** cervical cancer, uterine cancer, radical hysterectomy, lymphadenohysterocolpectomy, postoperative complications

## Abstract

Rationale The current standard surgical treatment for the cervix and uterine cancer is the radical hysterectomy (lymphadenohysterocolpectomy). This has the risk of intraoperative accidents and postoperative associated morbidity.

Objective The purpose of this article is the evaluation and quantification of the associated complications in comparison to the postoperative morbidity which resulted after different types of radical hysterectomy.

Methods and results Patients were divided according to the type of surgery performed as follows: for cervical cancer – group A- 37 classic radical hysterectomies Class III Piver - Rutledge -Smith ( PRS ), group B -208 modified radical hysterectomies Class II PRS and for uterine cancer- group C -79 extended hysterectomies with pelvic lymphadenectomy from which 17 patients with paraaortic lymphnode biopsy . All patients performed preoperative radiotherapy and 88 of them associated radiosensitization.

Discussion Early complications were intra-abdominal bleeding ( 2.7% Class III PRS vs 0.48% Class II PRS), supra-aponeurotic hematoma ( 5.4% III vs 2.4% II) , dynamic ileus (2.7% III vs 0.96% II) and uro - genital fistulas (5.4% III vs 0.96% II).The late complications were the bladder dysfunction (21.6% III vs 16.35% II) , lower limb lymphedema (13.5% III vs 11.5% II), urethral strictures (10.8% III vs 4.8% II) , incisional hernias ( 8.1% III vs 7.2% II), persistent pelvic pain (18.91% III vs 7.7% II), bowel obstruction (5.4% III vs 1.4% II) and deterioration of sexual function (83.3% III vs 53.8% II). PRS class II radical hysterectomy is associated with fewer complications than PRS class III radical hysterectomy , except for the complications of lymphadenectomy . A new method that might reduce these complications is a selective lymphadenectomy represented by sentinel node biopsy . In conclusion PRS class II radical hysterectomy associated with neoadjuvant radiotherapy is a therapeutic option for the incipient stages of cervical cancer.

Abbreviations: PRS- Piver Rutledge-Smith, II- class II, III- class III

## Introduction

Cervical and uterine cancers are the most common cancers of the genital area, being responsible for a high mortality among women. Cervical cancer is the fourth leading cause of female mortality with malignant neoplastic disease. Unfortunately Romania ranks first in Europe in terms of incidence (23.9 / 100,000) and mortality (11.8/100.000) of cervical cancer [**1**]. In developed parts of the world where screening programs have been implemented, the number of cases and deaths of cervical cancer have declined.

 The natural evolution of these cancers is characterized by local invasion through interstitial spaces of the pelvic connective tissue and by circulatory pathway of lymph dissemination through tributary limphnodes of uterus and cervix. Not often it can disseminate through anatomical pathways (salpingitis , vagina), blood (distant metastases) and nerve sheaths.

 Radical surgery in cancer has two major components represented by a wide ablation of the uterus with the nearby tissues and by regional lymphadenectomy in lymphofile cancers. This last procedure is performed for both diagnostic and therapeutic reasons. Radical hysterectomy (lymfadenohysterocolpectomy), which is the current standard surgical treatment, has the risk of intraoperative accidents and is associated with postoperative morbidity. The aim of this paper is to evaluate and to quantify these complications in comparison to postoperative morbidities of different types of radical hysterectomy.

 Group of female patients

 From January 2008 to December 2011 in the Department of Surgical Oncology II, on a prospective cohort, from Institute of Oncology "Prof. Dr. Al. Trestioreanu " Bucharest were performed 508 radical surgeries for cervical and endometrial cancer. Information taken from patient records or surgical operations and collected using the telephone interview, were analyzed and processed. 324 patients were evaluated from the total of 508, 184 being excluded because they were not eligible. Patients were divided according to the type of surgery performed and the stage of the disease, as follows: for cervical cancer group A -37 classical radical hysterectomies (18 std IIIB, 13 std IIIA, 6 std IIB ) and group B -208 modified radical hysterectomies (3 std IIIA , 87 std IIB, 118 std IIA) and for uterine cancer group C -79 extended hysterectomies with pelvic lymphadenectomy, from which 17 patients suffered a para-aortic lymphnode biopsy . All patients were preoperatively irradiated with a total dose range from 50 to 60 Gy which depended on the presence of local invasion and pelvic adenopathies, divided into several sessions.

 Description of the surgery operation

 Median pubo - supraumbilical laparotomy bypass the navel to the left and the inspection of the intra-abdominal organs. Exploring the peritoneal cavity with evaluation of local and distant desease extension and assessment of the operability state and the peritoneal lavage cytology. Biopsy of para-aortic adenopathies is assessed with the extemporaneous anatomo-pathological test. The anterior leaf of broad ligament is incised till we reach the round ligaments folds, afterwards we follow the ligation of the round ligaments. The radical hysterectomy is associated with bilateral anexectomy (adnexa are conserved in young patients which are unsuitable for radiotherapy). Consequently, the bilateral identification of ureters and then ligation of lumbo-ovarian ligaments. Dissection of the subperitoneal space and identification of the external iliac artery and vein is presented. Lymph nodes dissection and excision of tissue around the external iliac pedicle and common iliac bifurcation are identified. Lateral limit of dissection is given by genitofemural nerve. Posterior limit of dissection and excision of obturator fossa lymph nodes is given by obturatory nerve. Then we proceed according to the disease stage with classical radical hysterectomy (class III Piver-Rutledge-Smith [**3**]) - uterine artery ligation at its origin, uncrossing the uterine artery from the ureter, dissection of pelvic ureter untill it reaches the bladder; this three steps have the objective to do a resection of parameter as wide of its possible or modified radical hysterectomy(class II PRS ) - ureters are not released from pubo – vesical ligament and uterine artery ligation is made medial to the ureter. In endometrial cancers it is mandatory to do a para-aortic adenopathy biopsy. [**4**] The vagina is closed with separate absorbable suture wires in "X". The pelvis is not peritonized . 

## Results

The 37 patients with cervical cancer from group A were diagnosed with LLETZ (large loop excision of the transformation zone). Group B was formed by 208 patients with cervical cancer, 8% being diagnosed with the help of the biopsy forceps – punch biopsy, 62% by LLETZ and 30% by cervical conization. The average age of the pacients with cervical cancer was 52 years old, the youngest patient being 28 years old and the oldest 73 years old. The main histopathologic type was squamous carcinoma with 86% patients having the related subtypes: large-cell non keratinized carcinoma (67%), large cell keratinized carcinoma (17%), small cell carcinoma(2%) and adenocarcinoma with 14% pacients having the related subtypes: adenosquamos carcinoma (1%), mucinous adenocarcinoma (3%), endometrial adenocarcinoma(1%), pure adenocarcinoma (9%).

 79 patients with endometrial cancer from group C were diagnosed by biopsy curettage. In the endometrial cancer group the average age was 64 years old, the youngest being 46 while the oldest 83. The histopathological type which was identified in endometrial cancer was endometrioid adenocarcinoma, being present in all 79 patients.

 During these radical surgery operations accidental damage of the small intestine or the colon have occured in 5 cases (2%), the bladder was injured in 12 cases (4.8%), the ureter was injured in 3 cases (1.2%), and external iliac vein injury accompanied by massive hemorrhage occurred in 2 cases (0.8%). Most cases (n = 15) of intra-operative accidents have occurred during radical hysterectomy PRS III. All intraoperative complications were identified and resolved in the same surgical session; their discharge occurred at a variable time 5-14 days with an average of 10 days.

 Postoperative complications might be divided according to the period from occurring in early and late complications. The early complications have occurring within 7 days of surgery. They were urogenital fistula ( 5.4 % III PRS vs 0.96 % II PRS) , dynamic ileus (2.7 % III PRS vs 0.96 % II PRS ) supra-aponeurotic hematoma ( 5.4 % III PRS vs 2.4 % II PRS) and intra abominal bleeding ( 2.7 % III PRS vs 0.48 % II PRS ). It took 10 surgical reinterventions for these complications , 6 of them after radical surgery operation class III PRS and 4 after radical surgery operation Class II. In case of the extended hysterectomy associated with pelvic lymphadenectomy 4 of the patients (5% ) had dynamic ileus and one patient (1.3%) had required reintervention for intra-abdominal bleeding . The most common late complications were mild to moderate being represented by bladder dysfunction ( 21.6 % III PRS vs 16.35 % II PRS), lower limbs lymphedema(13.5 % III PRS vs 11.5 % II PRS) , urethral strictures (10.8 % III PRS vs 4.8 % II PRS ), incisional hernias ( 8.1 % III PRS vs 7.2 % II PRS ) , persistent pelvic pain ( 18.91 % III PRS vs 7.7 % II PRS ), bowel obstruction ( 5.4 % III PRS vs 1.4 % II PRS ). Among patients on which extended hysterectomy associated with pelvic lymphadenectomy is performed 10.1 % had mild bladder dysfunction,11.4 % lower limb lymphedema, 2.5 % urethral strictures , 3.8 % incisional hernias, 8.8% persistent pelvic pain, 1.3 % bowel obstruction.

 From the sex life point of view in group A (Class III PRS ) 55.2 % of patients state they have ceased their sex life years before surgery and have remained so after; in the subgroup of patients sexually active, after surgery 33.3 % suffered from dyspareunia, 50 % reported lack of sexual pleasure with thinning the frequency of intercourses and 16.6 % had a normal sex life. 40.85 % patients from group B relate they had stopped their sexual activity years before the modified radical hysterectomy (class II PRS ) remaining with the same status and after surgery. 28.2 % of sexually active pacients from group B had dyspareunia after surgery, 25.6 % reported lack of sexual pleasure with thinning frequency of intercourse and the remaining patients (46.2%) had been left with a normal sex life . In group C 78.5 % of patients said they haven’t had sex for years, with no change of this aspect postoperative. The reason of this huge percentage is the older age at which occurs the endometrial cancer. From the subgroup of patients which are still sexually active (n = 17), 29.4 % accuse dyspareunia after surgery and at 70.6% sex life had remained the same.

## Discussion

We have chosen Piver - Rutledge -Smith classification [**3**] of radical hysterectomy because it seemed the most explicit and closer to the types of surgeries we perform in Bucharest, Institute of Oncology "Prof. Dr. Al. Trestioreanu " .

 Urinary fistulas occurred after radical hysterectomy in 4 cases (3 cases of ureterovaginal fistulas and 1 case of vesicovaginal fistula). In their etiology there are two main components: surgery and pre-surgery radiotherapy, which acts complementary through the lesions produced at the uterine wall level, at the terminal ureter, at the vagina and at the pelvic tissue. Radical surgeries, which require, extensive dissection of the ureter and bladder, and can be determined through ischemic lesions, increase the risk of urogenital fistulas. Along with radical surgeries, there are also iatrogenic lesions (transfixiante wires which interest pelvic ureter and the posterior wall of the bladder) in the case of incomplete dissections which do not ensure good visibility and safe vaginal suture. The Urogenital fistulas diagnostic is a triad composed of: clinical examination (exam using valves and the probing of the 3 pads after taking blue methylene blue), cystoscopy and imaging examinations (iv urography/CT with contrast substances).

 It is essential to point out the fact that in the case of uretero -vaginal fistulas, the level diagnostic and the location of the fistula are very important (not always easy) to diagnose because they point out the best therapeutic procedure. These can be solved during the surgery when the diagnostic is made; in our experience they are accompanied by uretero hydronephrosis on the affected side, sometimes important (GRD III). The surgeries may consist in minimal invasive interventions (ureteral endoprosthesis Cook probe) or open uretero - vesical reimplantation , accompanied by dissolution of the fistula and resection of the affected portion of the ureter; these ones having better results being accomplished in all three cases.

 In case of vesico -vaginal fistula (cure of fistula using transabdominal procedures, transvesical or transvaginal) it is necessary at least 3 months after the onset of fistula, which is in fact the time required to the complete the delimitation of the devascularized areas and inflammatory remittance. It is found that these complications occur more frequently in PRS III radical hysterectomy ( 5.4 %) compared to PRS II (0.96 %). Our results in the case of PRS II radical hysterectomy are comparable with data from the literature (1.43 % Methfessel HD [**12**], 1.3% Hatck KD [**22**], 1 % Cai [**14**], 0.5 % Pikaart [**13**]).

 The dissection of the pelvic segment of the ureter during the radical hysterectomies may cause a variable level of ischemia which in time will cause segmental stenosis for the affected portion. A component which has high importance, is the radiotherapy that causes chronic and progressive damage to both pelvic fatty cellular tissue with extrinsec strangulation of the ureter , and also to the ureter wall, resulting into unilateral or bilateral uretero-hydronephrosis. Symptoms are often subtle, detection of the uretero-hydronephrosis is totally random, in general conducted with an ultrasound scan, but rarely can show signs of acute renal colic or sepsis (pyonephrosis). The treatment is minimally invasive (endoscopic) or surgical (uretero - vesical reimplantation), depending on the location and the extent of stenosis and on patient’s preference. Our data reveals a significant difference in occurrence of these complications between the two types of hysterectomy (10.45 % vs 4.28 % II III PRS PRS) , the results are similar to other studies from the literature (2% P.Benedetti Panici [**23**], 4.3 % Hatch KD [**22**]) .

 Bladder dysfunctions appear quite frequently after lymphadenohysterocolpectomy, a significant influence also having radiotherapy. The main dissatisfaction of patients is related to loss of urinating sensation and partially emptying the bladder. These complications occur because the pelvic plexus nerves are being sectioned (inferior hypogastric plexus). Parasympathetic nerves may be affected if the surgeon accidentally injures the bilateral sacral nerves during the surgical maneuvers or if the surgery is performed with a high degree of radicalism with wide excision of the paracervical, retroperitoneal and paracolpos tissue. Hypogastric sympathetic nerves can also be damaged during lymphadenectomy ilio - obturatory. Asynchrony between poor detrusor contraction and incomplete relaxation of the urethral sphincter leads to partial emptiness of the bladder. Patients who have lost in need urinating sensation are substituting it with other perceptions (distended abdomen, tension in the pelvic region, vague discomfort) which in time will be associated with a full bladder . They will learn to empty the bladder at an equal time interval using compensatory mechanisms (muscle strain with increased abdominal pressure). Some patients require intermittent urinary catheters. Most common symptoms in the deficit of emptying the bladder are dysuria, the vesical residue, overflow urine incontinence and frequent urinary tract infections .Functional incontinence is another cause of stress to patients . The limited deficit of urethral orifice but also the sympathetic nerve loss due to extensive resection of the parameters of the upper vaginal thirds can cause this complication. There are theories which say that in the physiology of this condition the main role could actually be considered the urethral sphincter. After 6-12 months most patients do not accuse any symptomatology, this occurs not due the improving of bladder function, but because of the means of compensation. To avoid these complications, lymphadenohysterocolpectomy techniques were imposed to preserve the autonomic nervous system but with the same level of radicalism. Urinary dysfunctions occur more rarely in case of class II radical hysterectomy PRS (16.35 % II vs 23.9 % III), the results are comparable with the data from literature (12.9 % Cai [**14**];14.5% Manchana [**18**], 7.5 % Pikaart [**13**], 10 % Wu [**42**] ;14.7% Landoni [**43**], 9.2 % Abrao [**44**], 16.2 % Ayhan [**45**]).

 Physiological postoperative ileus usually lasts for 2-3 days. This is caused by opening the peritoneal cavity, water- electrolyte imbalances and possible infections; its duration varies and is based on the duration of the surgery, the level of intestine manipulation and the dose of opioid used for anesthesia. When the transit for gas and feces is not resumed after a period longer than the one mentioned, then it is the case of paralytic ileus. There can happen severe complications with multiple consequences : increased post-surgery pain , increased episodes of nausea and vomiting , delayed oral feeding , poor wound healing, difficult mobilization, pulmonary complications, contact other nosocomial infections , prolonged hospitalization. In our study the presence of paralytic ileus is of 3% PRS III vs 1.17 % PRS II appeared less frequently than in the literature (10.5 % Fujita K [**16**]) post-surgery mechanical ileus occurs much later, after the onset of normal transit.

 Lower limb lymphedema is caused by the accumulation of lymph in the subcutaneous tissue after reducing lymphatic circulation. The blockage prevents lymph fluid to drain and thus the member gradually increases in thickness reaching in rare cases to elephantiasis associated with functional impotence. Lymphedema most often is unilateral, only in rare cases, it affects both limbs. An ilio - obturatory extended lymphadenectomy associated with radiotherapy increase the risk of lower limb lymphedema. It was found that for unknown reasons the lower limb lymphedema has a much lower frequency than upper limb lymphedema after radical mastectomy. In our study we found that a conservative surgery in terms of the level of resection of the parameters did not significantly reduce the incidence of lower limb lymphedema because the technique of lymphadenectomy did not modify in the two types of surgeries. So at the moment there is no cure for this complication. Patients have to protect the legs of any kind of infection through a rigorous local sanitary procedure and spare them as much to avoid prolonged orthostatism. Our results on the incidence of lymphedema ( 11.94 %III PRS vs 12.23 % II PRS ) are very similar to other studies from the literature ( T. Manchana [**18**] 2.1 %; K Bergmark [**17**] 25 %).

 Mechanical bowel obstructions occur due to postoperative fibrin adhesions which form a month after surgery but also because of fibrosis caused by radiotherapy. Intestinal obstruction can occur anytime from one month to more than 10 years after surgery after formation of the adhesions. These usually form between the greater omentum and parietal surgical scar, in a smaller number of cases may arise by fixing the omentum in the pelvis or between omentum and the sigmoid colon loop, between intestinal ansae and parietal surgical scar, by fixing the bowel in the pelvis and between each other intestinal ansae. The physiopathology of the fibrin adhesions formation involves the intraoperative trauma of the peritoneal leaves associated with low levels of tissue plasminogen activating factor (tPA). Correction of fibrinolysis by experimental administration of intraperitoneal tPA demonstrated a possibility of prophilaxy adhesions . Diagnosis of intestinal obstruction is based on classic symptoms: intermittent abdominal cramps , vomiting, bloating , constipation and a lack of gas if the intestine is completely blocked or diarrhea in case of partial obstruction. The suspicion of mechanical occlusion must be confirmed using Rx or CT. The only treatment is the surgery of the intestinal obstruction. The survey data shows that occlusion is a rare complication in the case of class II PRS (4.47% II PRS vs 1.55% III PRS): these findings beeing similar to the ones in the literature (5% Monk [**37**], 1.3% Magrina [**35**]).

 The incisional hernia is a subcutaneous protrusion of the organs from the peritoneal cavity made through a parietal defect formed after surgery. May be due to the surgical act or the patient's biological terrain. Parietal suture technichal errors and postoperative infection are directly related with the surgical act. Median subumbilical laparotomy is frequently followed by the appearance of incisional hernia because of the abdominal wall structure (Spiegel line; rectus abdominis sheath, direction and sense of force lines acting on the scar ). Increased abdominal pressure immediately after surgery may be a pathogenic factor for the occurrence of incisional hernia: urinary retention, vomiting , coughing, paralytic ileus. Factors related to the patient's biological terrain are: cancer, cirrhosis , obesity, diabetes mellitus, chronic lung disease, constipation, dysuria. The treatment is surgical and consists of incisional hernias cure with alloplastic material .

 Sexual function after radical hysterectomy is deteriorating because of physical reasons but mostly psychological. The most common side effects of the surgery are sexually vaginal dryness , dyspareunia and difficulty reaching vaginal orgasm. Vaginal dryness occurs due to lower estrogen levels after bilateral ovariectomy. Without this hormone vaginal lining becomes thinner and loses its elasticity, vaginal atrophy contributing to pain during intercourse. Low levels of sex hormones lead to lower libido. These consequences of radical hysterectomy can be treated by hormone replacement therapy or non-hormonal vaginal moisturizers and water-based lubricants. Replacement therapy: women who seek hormone replacement therapy have to check their breasts annualy by doing an ultrasound and / or a mammography due to elevated levels of estrogen in order to increase the risk of breast cancer. Another rare complication is loosing sensitivity of vagina and clitoris because of erigentes nerve damage (parasympathetic nerves with the origin in pelvic splanchnic nerves), involved in arousal and triggering the orgasmic response . Women reach orgasm very difficult or not at all and for them sex became a mechanical act whose only purpose is to pleasure their partner . Our study demonstrates that this complication occurs quite often; the frequency is in direct relation to the degree of radicality of lymphadenohysterocolpectomy ( 25.6 % PRS II vs 50 % PRS III). The same happens to women who could only reach orgasm through stimulation of the cervix during deep penetration; but after surgery pleasure during intercourse diminishes considerably.

 Although after surgery most women remained with a shorter vagina and sutured in cul-de-sac, they can have a normal sex life because the clitoris remains free and the vaginal mucosa remains sensitive. Sometimes in the first months after surgery, vaginal shortening can cause dyspareunia, but with local treatment, hormone replacement therapy and maintenance of sexual intercourse this problem disappears in time. Unfortunately there are a number of women who give up practicing sexual intercourse because of psychological shock that they had when they discovered the genital cancer; depression, anger, helplessness, sadness, fear, frustration are states of mind which contribute in time to the extinguish of sexual desire.

 In our study a considerable number of patients reported that they ceased having sex years before surgery (55.2% group A, group B 40.85%, 78.5% group C). Those, sexually active, who were operated by classical radical hysterectomy accused after the surgery dyspareunia, vaginal dryness, decreased libido to lack of sexual desire and the inability to have an orgasm. Patients at which was performed modified radical hysterectomy (Class II PRS) had a much lower percentage of problems in sexual life, paradoxically some of them declare that there was an improvement in the quality of sexual life. The same results were also obtained in a study conducted in Rome (Plotti F [**38**]), the literature is quite poor regarding the information on the subject.

 Radicality is a key feature of cancer surgery involving complete excision margins in healthy tissue of malignant lesions in order to control local and lymph invasion. The extent of surgery depends on with the: disease stage, type of cancer, histo-pathologic differentiation grade, patient age, associated diseases. Often, the surgeon is put in a difficult situation: on the one hand, he had to choose the grade of radicality of the operation he will make; on the other hand the associated risks and the patient's quality of life. As our study demonstrates the postoperative complications rise with the grade of radical surgery. Often a high degree radical operation with a high morbidity can be compensated with neoadjuvant treatment associated with more conservative surgery; so it has to be found an ideal formula for each individual oncologic case.

 Net advantage of modified radical hysterectomy is a considerable decrease of complications with a low risk of disease recurrence when surgery is preceded by neoadjuvant radiotherapy . Most patients who underwent modified radical hysterectomy enjoy a normal sex life because of the small damage risk of the erectile nerves and the excision of a small portion of the vagina. Decreased risk of complications is due to a less aggressive dissection of the ureters , a more conservative resection of cardinal ligaments and paracolpos and last but not least a minimum damage of inferior hypogastric plexus. Unfortunately modified radical hysterectomy does not solve the most common complication after lymphadenectomy namely lower limb lymphedema. A new method under study which might reduce complications of ilio - obturatory lymphadenectomy (often useless in less advanced cases) is selective lymphadenectomy with sentinel lymph node biopsy [**47**]. This involves injecting perilesional a radioisotope, then identification of sentinel lymph node intraoperative using the gamma camera, excision of it and intraoperative histologic examination [**46**]. If the sentinel node is not invaded theoretically means that no other node in the lymphatic basin is invaded and there is no need for lymphadenectomy. If the sentinel node is invaded pelvic lymphadenectomy should be performed to excise the potential positive nodes. If this technique will be standardized as in breast cancer and melanoma [**48**] will entail selective lymphadenectomy in cervical cancer and uterine body.

 These good results (with low complication rate) similar to the best results in the literature are due to the collective surgical experience, the large number of cases treated and modern attitude to approach the genital cancers.

## Conclusions

Lymphadenohysterocolpectomy and extended hysterectomy with ilio-obturatory lymphadenectomy and para-aortic biopsy are associated with considerable morbidity, which may increase the rate noticeably when surgical technique is poor. Morbidity rate (28.3%) in our study is comparable with data from the literature. PRS class II radical hysterectomy is associated with fewer complications than PRS class III, so it is a better therapeutic option when it is preceded by neoadjuvant radiotherapy for less advanced cancers.

 "Acknowledgement: This paper is supported by the Sectoral Operational Programme Human Resources Development (SOP HRD) 2007-2013, financed from the European Social Fund and by the Romanian Government under the contract number POSDRU/107/1.5/S/82839"
